# Supplementary material for: L-Arginine and asymmetric dimethylarginine (ADMA) transport across the mouse blood-brain and blood-CSF barriers: Evidence of saturable transport at both interfaces and CNS to blood efflux
Source: PLoS One. 2024 Oct 24;19(10):e0305318. doi: 10.1371/journal.pone.0305318 (PMC11501026; doi:10.1371/journal.pone.0305318)
Supplement: S1 Fig — Uptake is expressed as the percentage ratio of tissue to plasma (mL.100 g-1). Perfusion fluid contained either [3H]-arginine and [14C]-sucrose (open markers) or [3H]-ADMA and [14C]-sucrose (filled markers). Each point represents the mean ± SEM of 4–7 animals (GraphPad Prism 6.0 for Mac). (PDF) [file pone.0305318.s001.pdf]

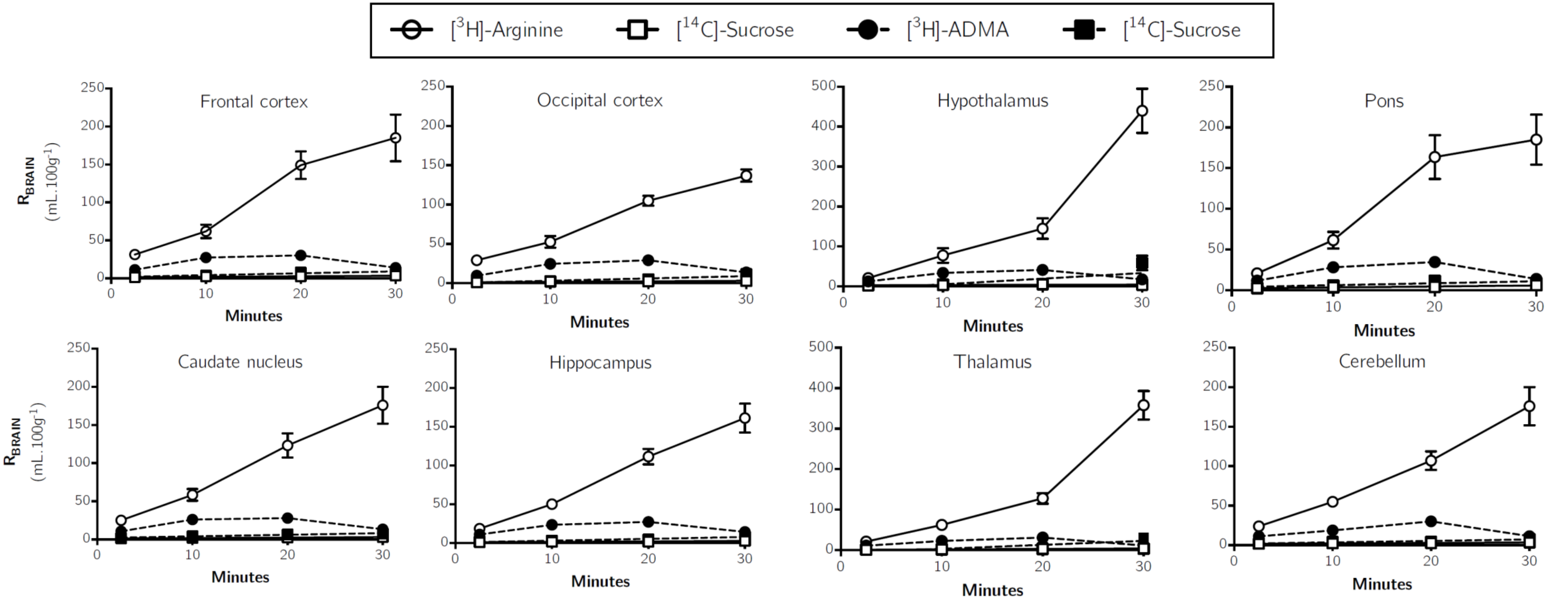


**S1 Fig**: **Comparative uptake of [$^3$H]-arginine, [$^3$H]-ADMA and [$^{14}$C]-sucrose as a function of time measured by *in situ* brain perfusion in anaesthetized mice.** Uptake is expressed as the percentage ratio of tissue to plasma (mL.100 g$^{-1}$). Perfusion fluid contained either [$^3$H]-arginine and [$^{14}$C]-sucrose (open markers) or [$^3$H]-ADMA and [$^{14}$C]-sucrose (filled markers). Each point represents the mean ± SEM of 4-7 animals (GraphPad Prism 6.0 for Mac).
