# Supplementary material for: L-Arginine and asymmetric dimethylarginine (ADMA) transport across the mouse blood-brain and blood-CSF barriers: Evidence of saturable transport at both interfaces and CNS to blood efflux
Source: PLoS One. 2024 Oct 24;19(10):e0305318. doi: 10.1371/journal.pone.0305318 (PMC11501026; doi:10.1371/journal.pone.0305318)
Supplement: S2 Fig — Uptake is expressed as the percentage ratio of tissue to plasma (mL.100 g-1). Each point represents the mean ± SEM of 5 animals. Kin and Vi values were determined as the slope and ordinate intercept of the computed regression lines (GraphPad Prism 6.0 for Mac). (PDF) [file pone.0305318.s002.pdf]

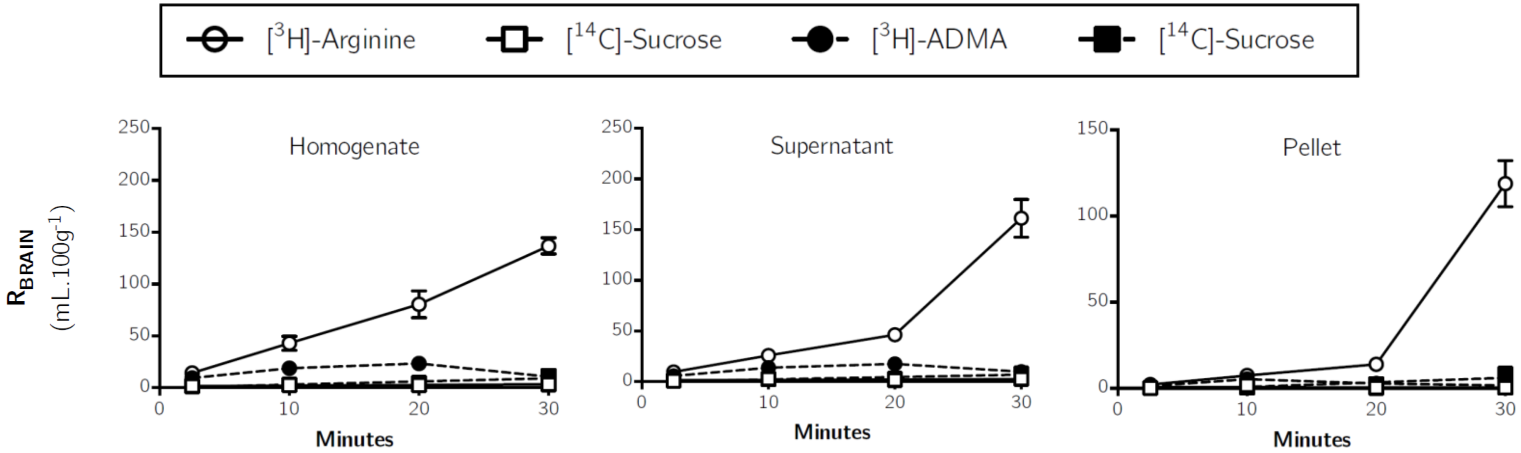

S2 Fig: Distribution of [^{3}H]-arginine, [^{3}H]-ADMA and [^{14}C]-sucrose in capillary depletion samples as a function of time. Uptake is expressed as the percentage ratio of tissue to plasma (mL.100 g^{-1}). Each point represents the mean ± SEM of 5 animals. K_{in} and V_i values were determined as the slope and ordinate intercept of the computed regression lines (GraphPad Prism 6.0 for Mac).
