# Supplementary material for: L-Arginine and asymmetric dimethylarginine (ADMA) transport across the mouse blood-brain and blood-CSF barriers: Evidence of saturable transport at both interfaces and CNS to blood efflux
Source: PLoS One. 2024 Oct 24;19(10):e0305318. doi: 10.1371/journal.pone.0305318 (PMC11501026; doi:10.1371/journal.pone.0305318)
Supplement: S3 Fig — Uptake is expressed as the percentage ratio of tissue or CSF to plasma (mL.100 g-1). Each point represents the mean ± SEM of 4–7 animals (GraphPad Prism 6.0 for Mac). (PDF) [file pone.0305318.s003.pdf]

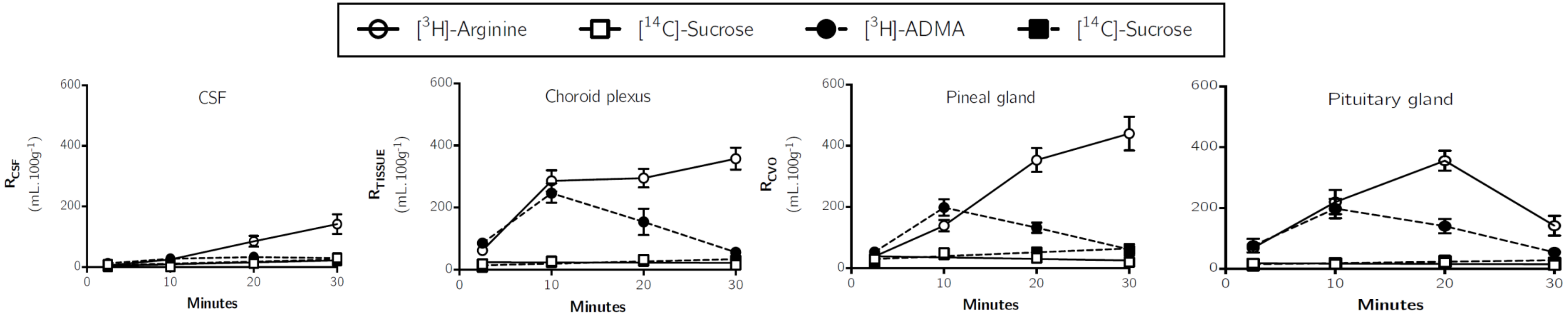

S3 Fig: Comparative distribution of [^{3}H]-arginine, [^{3}H]-ADMA and [^{14}C]-sucrose in the CSF, pineal gland, choroid plexus and pituitary gland following *in situ* brain perfusion as a function of time. Uptake is expressed as the percentage ratio of tissue or CSF to plasma (mL.100 g^{-1}). Each point represents the mean ± SEM of 4-7 animals (GraphPad Prism 6.0 for Mac).
