# Supplementary material for: L-Arginine and asymmetric dimethylarginine (ADMA) transport across the mouse blood-brain and blood-CSF barriers: Evidence of saturable transport at both interfaces and CNS to blood efflux
Source: PLoS One. 2024 Oct 24;19(10):e0305318. doi: 10.1371/journal.pone.0305318 (PMC11501026; doi:10.1371/journal.pone.0305318)
Supplement: S4 Fig — Uptake is expressed as the percentage ratio of tissue or CSF to plasma (mL.100 g-1). Brain samples have been corrected for [14C]-sucrose (vascular space). Perfusion time is 10 minutes. Each bar represents the mean ± SEM of 6–7 animals. (GraphPad Prism 6.0 for Mac). One-tailed unpaired Student’s t-test comparing means. *p < 0.05, **p < 0.01. (PDF) [file pone.0305318.s004.pdf]

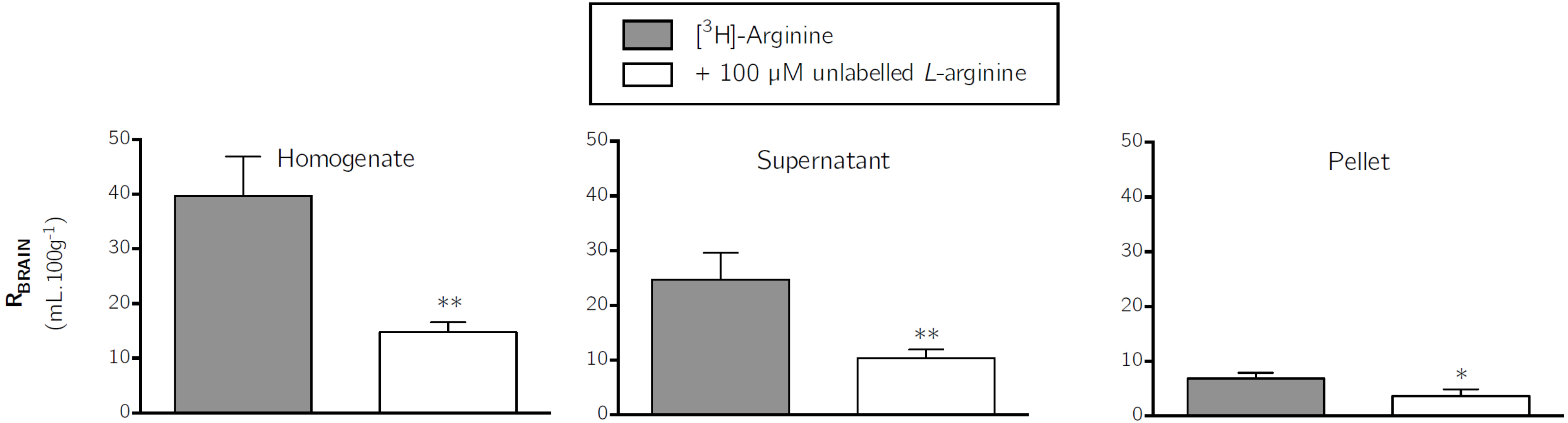

S4 Fig: The effect of 100µM un-labelled L-arginine on the distribution of [^{3}H]-arginine in capillary depletion samples. Uptake is expressed as the percentage ratio of tissue or CSF to plasma (mL.100 g^{-1}). Brain samples have been corrected for [^{14}C]-sucrose (vascular space). Perfusion time is 10 minutes. Each bar represents the mean ± SEM of 6-7 animals. (GraphPad Prism 6.0 for Mac). One-tailed unpaired Student's t-test comparing means. *$p < 0.05$, **$p < 0.01$.
