# Supplementary material for: L-Arginine and asymmetric dimethylarginine (ADMA) transport across the mouse blood-brain and blood-CSF barriers: Evidence of saturable transport at both interfaces and CNS to blood efflux
Source: PLoS One. 2024 Oct 24;19(10):e0305318. doi: 10.1371/journal.pone.0305318 (PMC11501026; doi:10.1371/journal.pone.0305318)
Supplement: S7 Fig — Uptake is expressed as the percentage ratio of tissue to plasma (mL.100 g-1). Perfusion time is 10 minutes. Each bar represents the mean ± SEM of 2–5 animals (GraphPad Prism 6.0 for Mac) for each region in the control group. The unlabelled ADMA group had varying numbers of experiments for example, CSF (n = 2), pineal gland (n = 3), choroid plexus (n = 4) and pituitary gland (n = 4) (GraphPad Prism 6.0 for Mac). Unpaired Student’s t-tests comparing means.*p<0.05, **p<0.01 and ***p < 0.001. (PDF) [file pone.0305318.s007.pdf]

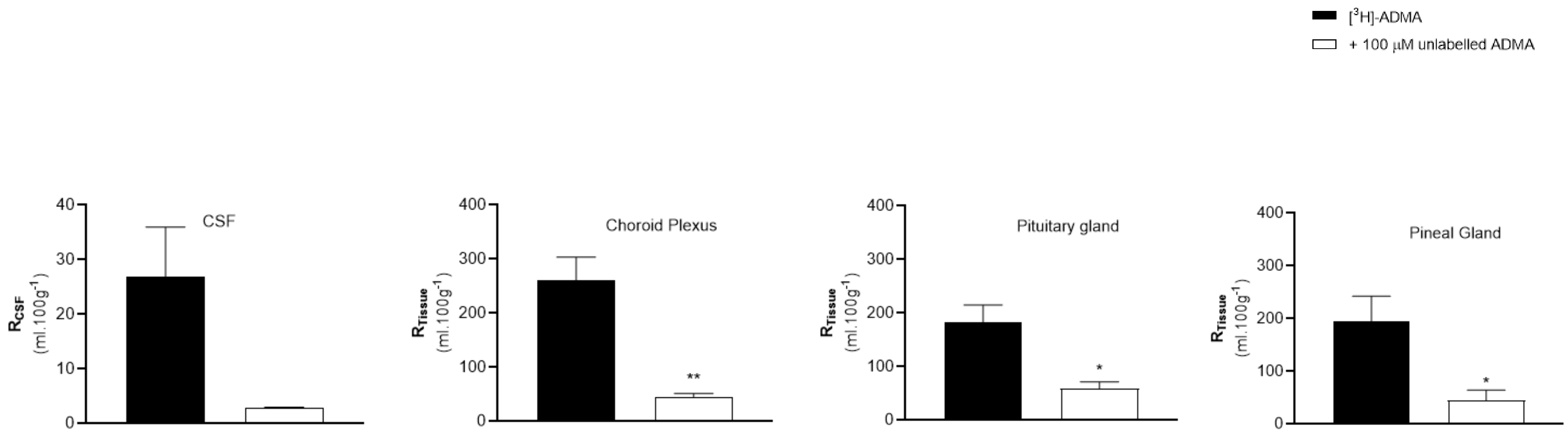

S7 Fig: **The effect of 100µM un-labelled ADMA on the uptake of [^{3}H]-ADMA in the CSF, choroid plexuses and circumventricular organs.** Uptake is expressed as the percentage ratio of tissue to plasma (mL.100 g^{-1}). Perfusion time is 10 minutes. Each bar represents the mean ± SEM of 2-5 animals for each region in the control group. The unlabelled ADMA group had varying numbers of experiments for example, CSF (n=2), pineal gland (n=3), choroid plexus (n=4) and pituitary gland (n=4) (GraphPad Prism 6.0 for Mac). Unpaired Student's t-tests comparing means.$^*p<0.05$, $^{**}p<0.01$ and $^{***}p < 0.001$.
