# Supplementary material for: L-Arginine and asymmetric dimethylarginine (ADMA) transport across the mouse blood-brain and blood-CSF barriers: Evidence of saturable transport at both interfaces and CNS to blood efflux
Source: PLoS One. 2024 Oct 24;19(10):e0305318. doi: 10.1371/journal.pone.0305318 (PMC11501026; doi:10.1371/journal.pone.0305318)
Supplement: S8 Fig — Uptake is expressed as the percentage ratio of tissue to plasma (mL.100 g-1) and is corrected for [14C]-sucrose (vascular space). Each bar represents the mean ± SEM of 5 animals (GraphPad Prism 6.0 for Mac). One-tailed unpaired Student’s t-test comparing means ***p < 0.001. (PDF) [file pone.0305318.s008.pdf]

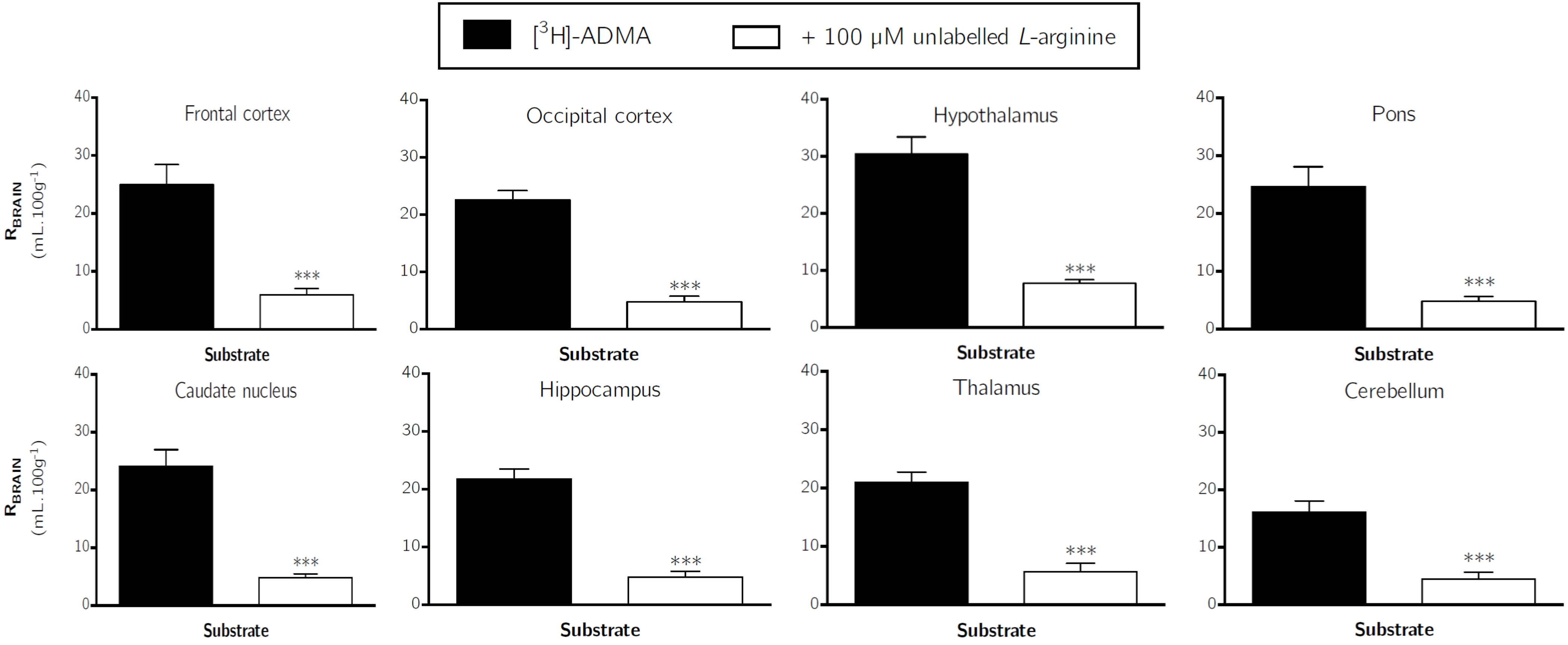

S8 Fig: The effect of unlabelled *L*-arginine on the regional brain uptake of [^{3}H]-ADMA (10 minute perfusion). Uptake is expressed as the percentage ratio of tissue to plasma (mL.100 g^{-1}) and is corrected for [^{14}C]-sucrose (vascular space). Each bar represents the mean ± SEM of 5 animals (GraphPad Prism 6.0 for Mac). One-tailed unpaired Student's t-test comparing means ***$p < 0.001$.
