# Supplementary material for: L-Arginine and asymmetric dimethylarginine (ADMA) transport across the mouse blood-brain and blood-CSF barriers: Evidence of saturable transport at both interfaces and CNS to blood efflux
Source: PLoS One. 2024 Oct 24;19(10):e0305318. doi: 10.1371/journal.pone.0305318 (PMC11501026; doi:10.1371/journal.pone.0305318)
Supplement: S9 Fig — Uptake is expressed as the percentage ratio of tissue to plasma (mL.100 g-1) and is corrected for [14C]-sucrose (vascular space). Each bar represents the mean ± SEM of 5 animals (GraphPad Prism 6.0 for Mac). Unpaired, one-tailed Student’s t-test was used to compare two means. **p<0.01 and ***p < 0.001. (PDF) [file pone.0305318.s009.pdf]

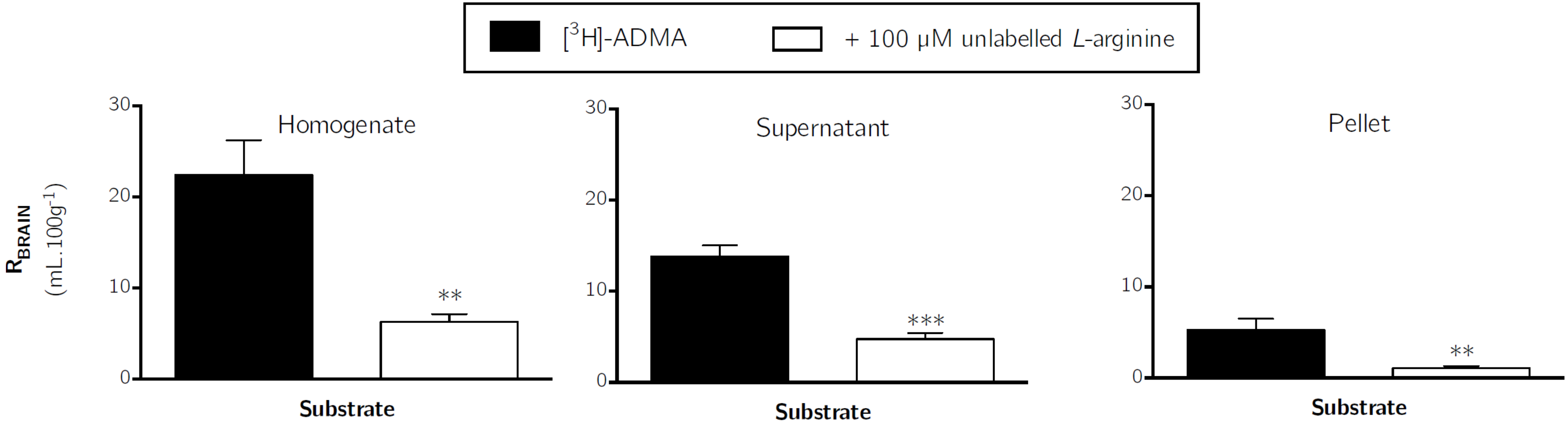

S9 Fig: The effect of unlabelled *L*-arginine on the distribution of [^{3}H]-ADMA in capillary depletion samples (10 minute perfusion). Uptake is expressed as the percentage ratio of tissue to plasma (mL.100 g^{-1}) and is corrected for [^{14}C]-sucrose (vascular space). Each bar represents the mean ± SEM of 5 animals (GraphPad Prism 6.0 for Mac). Unpaired, one-tailed Student's t-test was used to compare two means. **p<0.01 and ****p* < 0.001.
