# Supplementary material for: L-Arginine and asymmetric dimethylarginine (ADMA) transport across the mouse blood-brain and blood-CSF barriers: Evidence of saturable transport at both interfaces and CNS to blood efflux
Source: PLoS One. 2024 Oct 24;19(10):e0305318. doi: 10.1371/journal.pone.0305318 (PMC11501026; doi:10.1371/journal.pone.0305318)
Supplement: S10 Fig — Uptake is expressed as the percentage ratio of tissue or CSF to plasma (mL.100 g-1). Each bar represents the mean ± SEM of 5 animals (GraphPad Prism 6.0 for Mac). Unpaired, one-tailed Student’s t-test was used to compare two means. *p<0.05, **p<0.01 and ***p < 0.001. (PDF) [file pone.0305318.s010.pdf]

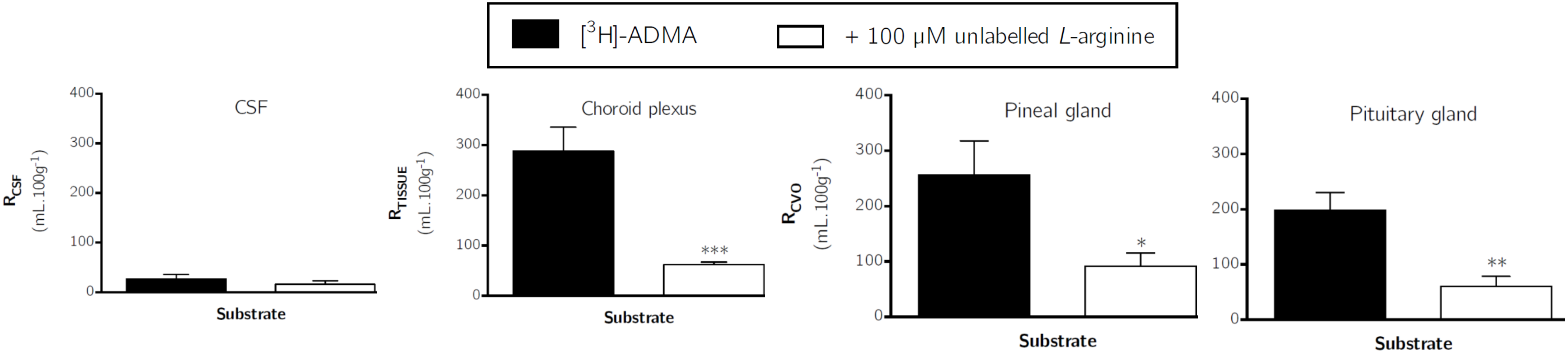

S10 Fig: The effect of unlabelled *L*-arginine on the distribution of [^{3}H]-ADMA in CSF, choroid plexus and CVOs (10 minute perfusion). Uptake is expressed as the percentage ratio of tissue or CSF to plasma (mL.100 g^{-1}). Each bar represents the mean ± SEM of 5 animals (GraphPad Prism 6.0 for Mac). Unpaired, one-tailed Student's t-test was used to compare two means. *p<0.05, **p<0.01 and ***p < 0.001.
