# Supplementary material for: L-Arginine and asymmetric dimethylarginine (ADMA) transport across the mouse blood-brain and blood-CSF barriers: Evidence of saturable transport at both interfaces and CNS to blood efflux
Source: PLoS One. 2024 Oct 24;19(10):e0305318. doi: 10.1371/journal.pone.0305318 (PMC11501026; doi:10.1371/journal.pone.0305318)
Supplement: S11 Fig — Uptake is expressed as the percentage ratio of tissue or CSF to plasma (mL.100 g-1). Each bar represents the mean ± SEM of 4–5 animals (GraphPad Prism 6.0 for Mac). One-way ANOVA with Dunnett’s post-hoc test comparing means to control ([3H]-ADMA only), ***p < 0.001). (PDF) [file pone.0305318.s011.pdf]

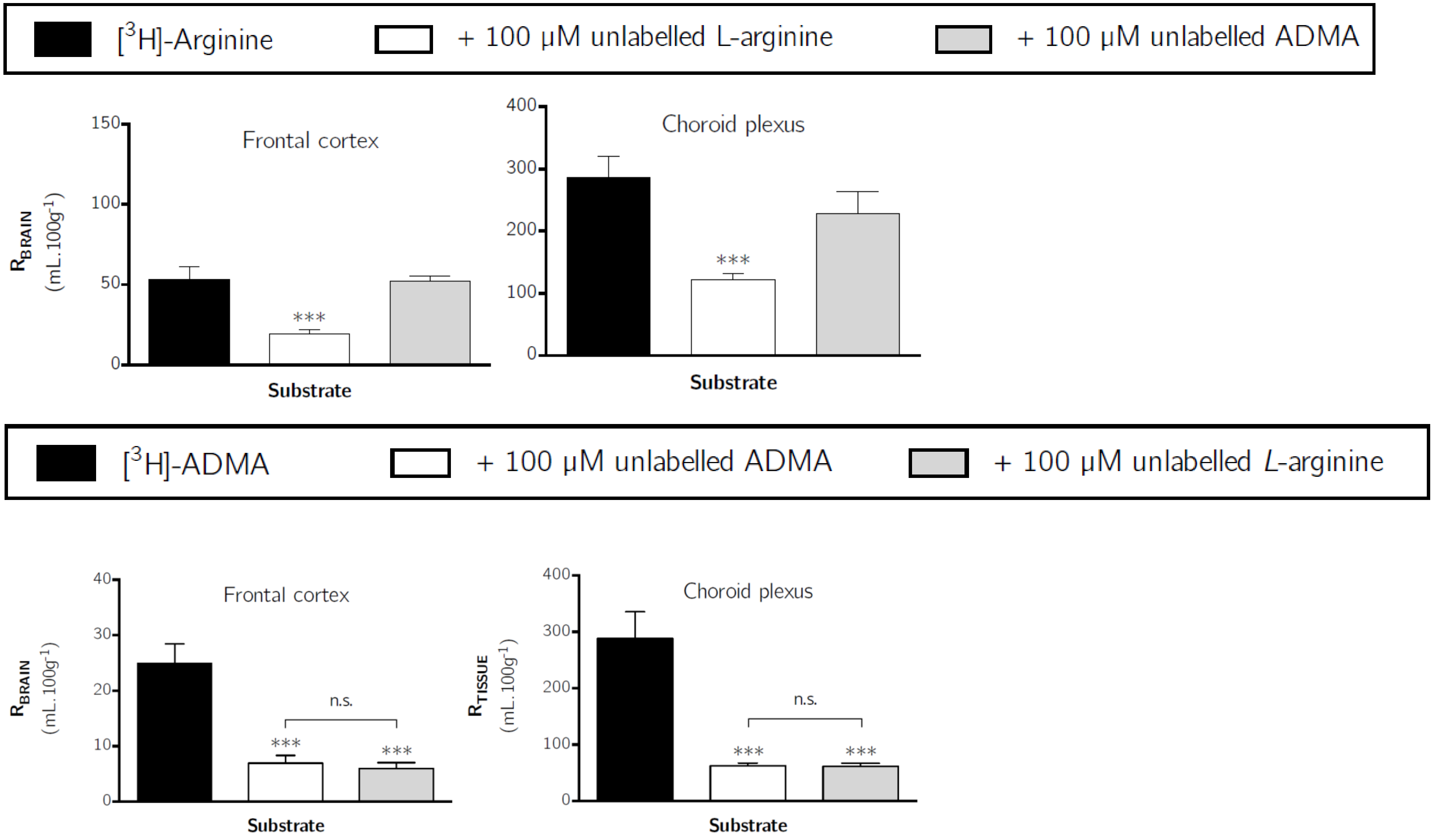

S11 Fig: Effect of either 100 µM unlabelled ADMA or 100 µM unlabelled *L*-arginine on the respective uptake and distribution of [^{3}H]-arginine or [^{3}H]-ADMA in the frontal cortex and choroid plexus. Uptake is expressed as the percentage ratio of tissue or CSF to plasma (mL.100 g^{-1}). Each bar represents the mean ± SEM of 4-5 animals (GraphPad Prism 6.0 for Mac). One-way ANOVA with Dunnett's post-hoc test comparing means to control ([^{3}H]-ADMA only), n.s. = not significant; ***$p < 0.001$).
