# Supplementary material for: L-Arginine and asymmetric dimethylarginine (ADMA) transport across the mouse blood-brain and blood-CSF barriers: Evidence of saturable transport at both interfaces and CNS to blood efflux
Source: PLoS One. 2024 Oct 24;19(10):e0305318. doi: 10.1371/journal.pone.0305318 (PMC11501026; doi:10.1371/journal.pone.0305318)
Supplement: Attachment — Submitted filename: Comments_29.06.2024.doc [file pone.0305318.s015.doc]

The author has done tremendous research on the CNS delivery of L-arginine and ADMA. Overall, the manuscript sounds good. Please, see the mentioned below comments-

1. Mechanism of ADMA Transport: While the study provides valuable data on ADMA and L-arginine distribution, it would be strengthened by delving deeper into the specific mechanisms of ADMA transport across the blood-brain barrier. Are there specific transporters involved? Is there evidence of active transport or passive diffusion?
2. Specificity of Arginine Supplementation: The study suggests arginine supplementation as a potential therapeutic strategy. However, it would be beneficial to explore the specificity of this approach. Does arginine supplementation primarily increase L-arginine levels or also affect ADMA levels? Could this approach lead to unintended consequences?
3. Clinical Relevance: The study highlights the importance of ADMA and L-arginine in vascular health. It would be valuable to discuss the clinical implications of the findings. How could these findings be translated into potential diagnostic or therapeutic strategies for patients with vascular diseases?
4. Comparison to Other Studies: The authors could strengthen their discussion by comparing their findings to existing literature on ADMA and L-arginine transport in the brain. Are there any discrepancies or areas of agreement with previous studies?
5. Limitations of the Study: The authors should acknowledge the limitations of their study, such as the use of animal models and the potential for extrapolation to human physiology. This would enhance the transparency and credibility of their research.
6. Future Directions: The study concludes with a call for further research. The authors could expand on this by outlining specific future directions for their research, such as investigating the role of specific transporters, exploring the effects of different arginine supplementation strategies, or conducting clinical trials to assess the therapeutic potential of their findings.
7. Statistical Analysis: The authors should provide more details about the statistical analysis used in their study. This would enhance the rigor and reproducibility of their findings.
8. Data Availability: The authors should clearly state the availability of their data, including the raw data and any supporting information. This would promote transparency and allow other researchers to replicate and build upon their findings.
9. Ethical Considerations: The authors should address any ethical considerations related to their study, particularly if they involved animal models. This would demonstrate their commitment to responsible research practices.
10. Clarity and Conciseness: The authors could improve the clarity and conciseness of their writing. This would make their findings more accessible to a wider audience.
11. Please improve the picture images of all, as the image is not in the higher pixel, and the image is getting blurred.
