# Supplementary material for: L-Arginine and asymmetric dimethylarginine (ADMA) transport across the mouse blood-brain and blood-CSF barriers: Evidence of saturable transport at both interfaces and CNS to blood efflux
Source: PLoS One. 2024 Oct 24;19(10):e0305318. doi: 10.1371/journal.pone.0305318 (PMC11501026; doi:10.1371/journal.pone.0305318)
Supplement: Attachment — Submitted filename: Responses to reviewers.docx [file pone.0305318.s016.docx]

**Manuscript number: PONE-D-24-21535**

**L -Arginine and asymmetric dimethylarginine (ADMA) transport across the mouse blood-brain and blood-CSF barriers: evidence of saturable transport at both interfaces and CNS to blood efflux.**

Dear Editors and Reviewers,

Thank you for taking the time to review this manuscript and provide constructive feedback.

Please find following our responses to the **editors and reviewers comments**:

**EDITOR REQUIREMENTS 1**

1. **Please ensure that your manuscript meets PLOS ONE's style requirements, including those for file naming. The PLOS ONE style templates can be found at
   https://journals.plos.org/plosone/s/file?id=wjVg/PLOSOne_formatting_sample_main_body.pdf and**
   [**https://journals.plos.org/plosone/s/file?id=ba62/PLOSOne_formatting_sample_title_authors_affiliations.pdf**](https://journals.plos.org/plosone/s/file?id=ba62/PLOSOne_formatting_sample_title_authors_affiliations.pdf)

**AUTHORS RESPONSE**

We have formatted the manuscript to ensure it meets all of PLOS ONE’s style requirements.

**EDITOR REQUIREMENT 2**

2. **In order to comply with PLOS ONE's guidelines for non-human primate experiments (http://journals.plos.org/plosone/s/submission-guidelines#loc-non-human-primates), please provide additional details regarding housing conditions, feeding regimens, environmental enrichment, and all relevant steps taken to alleviate suffering (anesthesia, analgesia, details about humane endpoints, euthanasia, etc.). Also indicate how often animal care staff monitored the health and well-being of the animals and the criteria used to make such assessments. Lastly, specify the disposition of animals at the end of the study (e.g. euthanasia, returned to home colony, etc.). If animals were euthanized following the study, please provide the method of sacrifice.**

**AUTHORS RESPONSE**

We did not use non-human primates in this study. However, we have provided additional details under the animals and anaesthesia section Lines 147-173.

**EDITOR REQUIREMENT 3**

**3. Please review your reference list to ensure that it is complete and correct. If you have cited papers that have been retracted, please include the rationale for doing so in the manuscript text, or remove these references and replace them with relevant current references. Any changes to the reference list should be mentioned in the rebuttal letter that accompanies your revised manuscript. If you need to cite a retracted article, indicate the article’s retracted status in the References list and also include a citation and full reference for the retraction notice.**

**AUTHORS RESPONSE:**

We confirm we have reviewed the reference list. As far as we are aware no article that we cite in this paper has, as yet, been retracted.

**REVIEWER 1:**

1. **REVIEWER 1 COMMENT:**

**- L65-70: Please cite necessary studies about this crucial topic.**

**AUTHOR RESPONSE:**

Original manuscript version: Lines 65-70 states this:

‘As *L*-arginine is the precursor for NO synthesis (7) and ADMA a potent endogenous inhibitor of NO synthesis (5) (6), the interplay between the transport of these two cationic amino acids at the blood-brain barrier (BBB) and blood-cerebrospinal fluid (CSF) barriers is likely to directly relate to NO production at these interfaces and within the central nervous system (CNS). In fact, the arginine/ADMA plasma concentration ratio is widely considered to be an important indicator of NO bioavailability’.

**REFERENCES**

(5) Vallance P, Leone A, Calver A, Collier J, Moncada S. Accumulation of an endogenous inhibitor of nitric oxide synthesis in chronic renal failure. Lancet. 1992 Mar 7;339(8793):572–5.

(6) Vallance P, Leone A, Calver A, Collier J, Moncada S. Endogenous Dimethylarginine as an Inhibitor of Nitric Oxide Synthesis. J Cardiovasc Pharmacol. 1992 Apr;20:S60–2.

(7) Palmer RMJ, Ashton DS, Moncada S. Vascular endothelial cells synthesize nitric oxide from L-arginine. Nature. 1988 Jun;333(6174):664–6.

We have now reworked and expanded this section by adding more citations (References 9,11 and 12) and referring to new studies (reference number 5, 9 and 10). The original version Lines 65-72- has now become revised version Lines 72-83 and states the following:

while the third isoform is inducible (iNOS). Asymmetric dimethylarginine (ADMA) is produced by all cells and is a product of protein degradation and competes with *L*-arginine for each of the three NOS isoforms (5). However, ADMA cannot be used as a substrate by NOS and as a result ADMA is a biologically significant inhibitor of NO production (6,7).

As *L*-arginine is the precursor for NO synthesis (8) and ADMA a potent endogenous inhibitor of NO synthesis (6)(7), the arginine/ADMA plasma concentration ratio is widely considered to be an important indicator of NO bioavailability (9,10). In addition, *L*-arginine and ADMA have been shown to influence their human brain endothelial and umbilical vein endothelial cellular availability via transport interactions (11)(12). Together these facts imply that the interplay between the transport of these two cationic amino acids at the blood-brain barrier (BBB) and blood-cerebrospinal fluid (CSF) barriers is likely to directly relate to NO production at these interfaces and within the central nervous system (CNS). Interestingly,

(5) Wilcken DEL, Sim AS, Wang J, Wang XL. Asymmetric dimethylarginine (ADMA) in vascular, renal and hepatic disease and the regulatory role of l-arginine on its metabolism. Mol Genet Metab. 2007 Aug;91(4):309–17.

(6) Vallance P, Leone A, Calver A, Collier J, Moncada S. Accumulation of an endogenous inhibitor of nitric oxide synthesis in chronic renal failure. Lancet. 1992 Mar 7;339(8793):572–5.

(7) Vallance P, Leone A, Calver A, Collier J, Moncada S. Endogenous Dimethylarginine as an Inhibitor of Nitric Oxide Synthesis. J Cardiovasc Pharmacol. 1992 Apr;20:S60–2.

(8) Palmer RMJ, Ashton DS, Moncada S. Vascular endothelial cells synthesize nitric oxide from L-arginine. Nature. 1988 Jun;333(6174):664–6.

(9) Bode-Boger, S.M.; Boger, R.H.; Kienke, S.; Junker, W.; Frolich, J.C. Elevated l-arginine/dimethylarginine ratio contributes to enhanced systemic NO production by dietary l-arginine in hypercholesterolemic rabbits. *Biochem. Biophys. Res. Commun.* **1996**, *219*, 598–603.

(10) Brinkmann SJ, Wörner EA, Buijs N, Richir M, Cynober L, van Leeuwen PA, Couderc R. The Arginine/ADMA Ratio Is Related to the Prevention of Atherosclerotic Plaques in Hypercholesterolemic Rabbits When Giving a Combined Therapy with Atorvastatine and Arginine. Int J Mol Sci. 2015 May 29;16(6):12230-42. doi: 10.3390/ijms160612230. PMID: 26035753; PMCID: PMC4490441.

(11) Shin S, Thapa SK, Fung HL. Cellular interactions between L-arginine and asymmetric dimethylarginine: Transport and metabolism. PLoS One. 2017 May 31;12(5):e0178710.

(12) Watson CP, Pazarentzos E, Fidanboylu M, Padilla B, Brown R, Thomas SA. The transporter and permeability interactions of asymmetric dimethylarginine (ADMA) and L-arginine with the human blood–brain barrier in vitro. Brain Res. 2016;1648:232–42.

**2. REVIEWER 1 comment**

**Original version Lines 127-147: Please provide references to support the advantages mentioned in the “in situ brain/choroid plexus perfusion” technique.**

**AUTHOR RESPONSE:**

We have now added the following references to support each advantage as now stated in the

revised version **Lines 183-210.**

These are:

Sanderson L, Khan A, Thomas S. Distribution of suramin, an antitrypanosomal drug, across the blood-brain and blood-cerebrospinal fluid interfaces in wild-type and P-glycoprotein transporter-deficient mice. Antimicrob Agents Chemother. 2007;51(9):3136–46.

Thomas Née Williams SA, Segal MB. Identification of a saturable uptake system for deoxyribonucleosides at the blood-brain and blood-cerebrospinal fluid barriers. Brain Res. 1996;741(1–2).

**3. REVIEWER 1 comment**

**- L163 (original version): How the CSF sample was taken is not clearly explained.**

**AUTHOR RESPONSE:**

We have now explained the procedure in more detail.

This can be found in the revised version **Lines 226-229** and is as follows:

At the set perfusion time a CSF sample was taken by inserting a pulled glass micropipette connected to silicon tubing and a syringe into the cisterna magna. Gentle suction was applied, and CSF was withdrawn. Only clear CSF samples were processed and taken for analysis. Samples contaminated with blood were discarded.

**3. REVIEWER 1 comment**

**- Could the authors explain why they chose to perform one-tailed student-t tests instead of two-tailed and two-tailed test for one analysis (L490)? What are the significant differences based on two-tailed test, are they the same or different from current differences? Two-tailed test might be more reliable in terms of significance validity.**

**AUTHOR RESPONSE:**

We have reviewed the statistical analysis performed on line 490 (original manuscript) for the comparison of [^3^H]arginine and [^3^H]ADMA and we can confirm that Student’s unpaired two tailed t-tests were performed to compare the two means at each time point (revised version Lines 605).

We chose not use a one tailed t-test as there was one lower value sample in the [^3^H]arginine group compared to the [^3^H]ADMA group samples at 10 minutes in most regions (except cerebellum).

**4. REVIEWER 1 comment**

**Discussion:**
 **- L648-xxx: The authors mention that there might be a concentration gradient favouring flux of [^3^H]-arginine from the brain back into the plasma, however, the plasma concentrations of L-arginine is different in humans and mice. The adaptation of the results to humans could be different, how do the authors explain that [^3^H]-arginine sequestration to brain would be similar in humans in that case?**

**AUTHOR RESPONSE:**

a)-As well as stating (revised version Lines 801-803) that the human and mouse plasma concentration of arginine is in the micromolar range (100 versus 140 μM, respectively) we now describe further on in the discussion (revised version L926 -L932) the kinetic constants for arginine transport in human and animal cells. They are similar in human and mouse cells. To do this we have also added references 52-54 listed below.

Revised version lines 926-932:

Furthermore, the human gene CAT-1 shares 87.6% amino acid identity with the mouse gene (52). Importantly, the high degree of sequence conservation has been shown to reflect functional similarity of the human transporters with their mouse homologues (53,54). *L*-arginine uptake by oocytes expressing hCAT-1 or mCAT-1 having a K_m_ of 110-160 μM and 140-250 μM respectively (53,54)(55)(56). This would further suggest that there would be a functional similarity of the human and mouse CATs.

(52) Yoshimoto T, Yoshimoto E, Meruelo D. Molecular cloning and characterization of a novel human gene homologous to the murine ecotropic retroviral receptor. Virology. 1991 Nov;185(1):10–7.

(53) Closs EI, Lyons CR, Kelly C, Cunningham JM. Characterization of the third member of the MCAT family of cationic amino acid transporters. Identification of a domain that determines the transport properties of the MCAT proteins. Journal of Biological Chemistry. 1993 Oct;268(28):20796–800.

(54) Closs EI, Gräf P, Habermeier A, Cunningham JM, Förstermann U. Human Cationic Amino Acid Transporters hCAT-1, hCAT-2A, and hCAT-2B:  Three Related Carriers with Distinct Transport Properties ^,^. Biochemistry. 1997 May 1;36(21):6462–8.

b) We have further clarified the following points (revised manuscript lines 916-926):

Interestingly, the cationic amino acids transport system, system-y^+^, has been detected at both luminal and abluminal membranes in brain capillary endothelial cells, with prevalence at the abluminal membrane (40). In addition, the half-saturation constants (K_m_) for arginine transport by murine CAT1 expressed in Xenopus oocytes and the y^+^-system expressed in human fibroblasts is 77 ± 2 μM and 40 ± 0.05 μM, respectively (32)(50)(49). It is therefore plausible that the transporters for arginine detected at the blood-CNS interfaces in our study are system-y^+^ transporters. We have previously identified the presence of the y^+^-system protein, CAT1, in human BBB cells (hCMEC/D3) using Western blotting and immunofluorescence (12)(51) and CAT 1 is expressed at the blood-CSF barrier specifically the rat choroid plexus (32)(41).

**5. REVIEWER 1 comment**

**- The changes of arginine concentrations might result in changes in ADMA concentrations endogenously in brain, rather than transport mechanisms, as they regulate one another’s metabolism. The opposite is also possible. What would be the authors’ comments about this? (Please see:** [**https://doi.org/10.1016/j.ymgme.2007.04.017**](https://doi.org/10.1016/j.ymgme.2007.04.017)**)**

**AUTHOR RESPONSE:**

We gave now added the following information to the manuscript:

**L1044 now states:**

Although it must be noted that other mechanisms may also affect intracellular ADMA concentrations and/or NO production. For example, arginine inhibits ADMA catabolism by competing with ADMA for dimethylarginine dimethylaminohydrolase (DDAH) (67) and….

**REVIEWER 2**

**Reviewer #2: The author has done tremendous research on the CNS delivery of L-arginine and ADMA. Overall, the manuscript sounds good. Please, see the mentioned below comments-

1. REVIEWER 2 comment: Mechanism of ADMA Transport: While the study provides valuable data on ADMA and L-arginine distribution, it would be strengthened by delving deeper into the specific mechanisms of ADMA transport across the blood-brain barrier. Are there specific transporters involved? Is there evidence of active transport or passive diffusion?**

**AUTHOR RESPONSE:**

**We have already explained that the self-inhibition studies indicated the presence of transporters for ADMA into the CNS.**

**Amended version lines 938-943:**

This suggests the use of transporters to transfer [^3^H]-ADMA across both mouse blood-brain and blood-CSF barriers. Importantly, this is the first study to reveal saturable transport of ADMA at the blood-CSF barrier (choroid plexus). It remains to be seen if these transporters are saturated by ADMA at physiological plasma concentrations which are 0.30-1.57 μM in humans (55) and 1.07-1.58 μM in mice (56).

**We have now also added the following information:**

**Amended version Lines 826-839.**

The concentration of [^3^H]-ADMA achieved in the CNS samples (except the choroid plexus, pineal and pituitary glands) was lower than that in the artificial plasma at all time points. This would indicate that transporters (and not just passive diffusion) were involved in the brain-to-plasma efflux of [^3^H]-ADMA. However, the CSF (choroid plexus)-to-blood transfer could involve transporters and/or just be passive diffusion of [^3^H]-ADMA.

**Amended version Lines 848-856.**

This would suggest that multiple saturable transport mechanisms with different kinetics are involved in ADMA transfer across cell membranes and/or that the transporter is bidirectional and is capable of transferring ADMA into and out of the cell with differing affinities. Interestingly, system y^+^ is a bidirectional uniporter and can transfer positively charged amino acids with a K_m_ for influx being lower than the K_m_ for efflux(42). The results so far described would indicate that [^3^H]-ADMA can cross the cerebral capillary endothelium (i.e. the site of the BBB) and the blood-CSF barrier in both directions (i.e. blood-to-CNS and CNS-to-blood) and that transporters are involved, at least in the brain-to-blood transfer.

**Amended version Lines 1084-1087**

In the future we hope to explore [^3^H]-arginine and [^3^H]-ADMA transport across the blood-brain and blood-CSF barriers in more detail determining the pharmacokinetic constants and the identity of the transporters involved. This may further contribute to understanding the contribution of ADMA efflux to any potential benefits of arginine supplementation.

**2. REVIEWER 2 comment: Specificity of Arginine Supplementation: The study suggests arginine supplementation as a potential therapeutic strategy. However, it would be beneficial to explore the specificity of this approach. Does arginine supplementation primarily increase L-arginine levels or also affect ADMA levels? Could this approach lead to unintended consequences?**

**AUTHOR RESPONSE:**

a) Revised manuscript L1017 we now have added the following information (see red):

This present study would support the theory that arginine supplementation can increase NO production not by inhibiting arginine influx, but by inhibition of [^3^H]-ADMA influx and/ or stimulating the efflux of [^3^H]-ADMA. This would ultimately lower the intracellular concentration of ADMA, but not affect the intracellular concentration of *L*-arginine significantly. The reasons for this are as follows. Firstly, our data supports the observations that the transporter for arginine would be operating at nearly maximum capacity in other words is nearly fully saturated (K*_m_* 25-77 μM) within the physiological plasma concentration range of endogenous arginine (human ~100 μM and mouse ~140 μM)(34) (33). This together with the facts that: (i) *L-*arginine transport by CAT-1 is a pre-requisite of NO production by the endothelium (62)(63) and ii) eNOS is normally already saturated (K*_m_* ~3-36 μM) with endogenous intracellular *L*-arginine (840±90 μM)(15)(16)(64) (63), would indicate that raising the plasma concentration of arginine would not increase NO production by increasing the intracellular concentration of arginine (65). Secondly, our data provides *in situ* evidence that ADMA can cross cell membranes using transporters, transport occurs in both directions and that arginine can reduce ADMA cellular accumulation. Thus, it is possible that supplementation with arginine would affect ADMA transport (efflux) resulting in the displacement of intracellular ADMA from eNOS (inhibition constant (K*_i_*) 0.9μM) (16) and increased NO production (55,66)(65).

b) We have now also expanded on this by adding information about the potential effects of arginine supplementation as follows: Revised manuscript L1035-1056:

Although a physiological intracellular concentration of ADMA of about 3.6 μM is thought to have only a modest effect (10%) on endothelial NO production (16). Pathophysiological plasma concentrations of ADMA, which are 3- to 9-fold higher, inhibit NO production by 30-70% (16). Studies in hypercholesterolemic young adults have demonstrated that arginine supplementation alleviates endothelial dysfunction (14). Although ADMA was not measured in this clinical study, hypercholesterolemia is associated with elevated plasma ADMA concentrations (2.17±0.15 μM versus 1.03±0.09 μM) (67). Overall, this suggests that arginine supplementation could affect ADMA transport (efflux) and consequently increase NO production particularly if ADMA plasma concentrations are higher than normal. Although it must be noted that other mechanisms may also affect intracellular ADMA concentrations and/or NO production. For example, arginine inhibits ADMA catabolism by competing with ADMA for dimethylarginine dimethylaminohydrolase (DDAH) (67) and ADMA may also increase the apparent K*_m_* of NOS for arginine (55,66). Indeed, arginine supplementation may cause multiple mechanisms to act in parallel. The relative importance of CAT, DDAH on intracellular ADMA concentrations and/or NO production depending on pathophysiological circumstance and ADMA plasma concentrations. This complex relationship may explain the absence of measurable clinical benefits of arginine supplementation in some clinical studies(5). It also suggests that more detailed consideration be given to the ADMA plasma concentration of patients before recommending arginine supplementation as a therapeutic strategy. Higher plasma concentrations of ADMA indicating that arginine supplementation would be more successful at increasing endothelial NO production and leading to beneficial effects.

3. **REVIEWER 2 comment: Clinical Relevance: The study highlights the importance of ADMA and L-arginine in vascular health. It would be valuable to discuss the clinical implications of the findings. How could these findings be translated into potential diagnostic or therapeutic strategies for patients with vascular diseases?**

**AUTHOR RESPONSE:**

As stated above we have now provided more information (Revised version Lines 1052 to 1054).

**4. REVIEWER 2 comment: Comparison to Other Studies: The authors could strengthen their discussion by comparing their findings to existing literature on ADMA and L-arginine transport in the brain. Are there any discrepancies or areas of agreement with previous studies?**

**AUTHOR RESPONSE:**

a) Revised version L76-83 (red sentences): We have now included the following information in the introduction:

As *L*-arginine is the precursor for NO synthesis (8) and ADMA a potent endogenous inhibitor of NO synthesis (6)(7), the arginine/ADMA plasma concentration ratio is widely considered to be an important indicator of NO bioavailability (9,10). In addition, *L*-arginine and ADMA have been shown to influence their human brain endothelial and umbilical vein endothelial cellular availability via transport interactions (11)(12). Together these facts imply that the interplay between the transport of these two cationic amino acids at the blood-brain barrier (BBB) and blood-cerebrospinal fluid (CSF) barriers is likely to directly relate to NO production at these interfaces and within the central nervous system (CNS).

b) In the discussion we have also now included the following sentence that refers to another study:

Revised version L 881: We have previously observed a greater ability (more than -three to -five-fold) of [^3^H]-arginine (7 nM) compared to [^3^H]-ADMA (38 nM) to cross brain capillary endothelial cell membranes by means of accumulation studies in hCMEC/D3 cells (12).

c) In addition, we have now referred to our earlier study on revised version line 888.

As arginine has a higher hydrophilicity when compared to ADMA and is of similar hydrophilicity to sucrose, the greater ability of arginine to accumulate within all regions of the brain *in situ* may be related to the use of specific transporters as also suggested by our previous *in vitro* study (12).

d) In addition, we have now referred to another study on revised version line 975:

In contrast to these cross-inhibition studies, the transport of a cationic amino acid analogue, [^3^H]-eflornithine (720 nM), into hCMEC/D3 cells appears much more sensitive to inhibition by 100 μM ADMA than 100 μM *L-*arginine (51).

e) And mentioned another study in the revised version lines 1011 to 1014:

Our studies confirm that the transport systems used by ADMA and *L*-arginine at the blood-CNS barriers appear to be shared to some degree, as they each affect the others' transport (Figs 10-12; Fig S8-10) and the transport of other cationic amino acids(51), but to a differing extent (Fig S11).

f) We would like to highlight that throughout the discussion we already refer to other studies including those that focus on brain transport of arginine and ADMA. For example, revised version L904-912: We already state:

Saturable transport of radiolabelled *L*-arginine has previously been observed across the rat BBB using the *in situ* brain perfusion technique (32)(43) and at the blood-CSF barrier using the isolated perfused sheep choroid plexus technique (44)(45). Although we did not determine the half-saturation constant (K_m_) of [^3^H]-arginine transport across the blood-CNS interfaces in our mouse study, our data would align with the half-saturation constant (K_m_) for arginine at the BBB previously determined using a similar method in rats (56 ± 9 μM) and the brain uptake index method in rats (40 ± 24 μM (±standard deviation))(43) and the blood-side (basolateral membrane) of the choroid plexus epithelium determined using the isolated perfused choroid plexus of the sheep (25.4 ± 5.1 μM) (32) (46).

g) **Revised manuscript L979-984: We also state:**

Interestingly, cross-competition studies revealed that [^3^H]-arginine uptake into the isolated incubated rat choroid plexus can be inhibited by another methylated arginine, N^G^-methyl-*L*-arginine, at a concentration of 500 μM (60). This *in vitro* method focuses on molecule movement across the apical/CSF side of the choroid plexus, in contrast to the luminal/blood side of the choroid plexus that is examined by the *in situ* brain/choroid plexus perfusion technique.

5. **REVIEWER 2 comment: Limitations of the Study: The authors should acknowledge the limitations of their study, such as the use of animal models and the potential for extrapolation to human physiology. This would enhance the transparency and credibility of their research.**
**AUTHOR RESPONSE:**

**We now state the following in the revised version Line 993-1009:**

Our study used the *in situ* brain/choroid plexus perfusion method in adult BALB/c mice to study the transport of [^3^H]-arginine and [^3^H]-ADMA across the BBB and blood-CSF barrier. Importantly, these barriers are very complex interfaces and cannot be replicated fully using cell culture methods. However, the *in situ* results are difficult to interpret conclusively due to multiple interacting factors such as: i) transport of the test molecule may occur into and out of the CNS (as suggested by the ADMA multiple time uptake data presented) ii) transfer of test molecules from brain tissue to CSF and CSF to brain tissue iii) loss of integrity of the radiolabels to the test molecule within the brain tissue and CSF and then removal of the radiolabel from the CNS iv) different transporters for the test molecule may be expressed at the BBB and blood-CSF barrier. Despite these limitations, consideration of all these facts still allows important conclusions to be drawn from the results. In addition, the data obtained from these *in situ* studies can be compared to data obtained *in vitro* as a means of further validating the methods and data. The characteristics of mouse models are of interest due to the availability of transgenic animals, oligoprobes, antibodies and mouse models of disease. Thus, expansion of our knowledge of mouse cells is of relevance to scientific investigations. Furthermore, mice are ideal for these studies as their BBB is similar in structure and function to that of humans.

6. **REVIEWER 2 comment: Future Directions: The study concludes with a call for further research. The authors could expand on this by outlining specific future directions for their research, such as investigating** **the role of specific transporters, exploring the effects of different arginine supplementation strategies, or conducting clinical trials to assess the therapeutic potential of their findings.**

**AUTHOR RESPONSE:**

As well as suggesting on line 1052:

that more detailed consideration be given to the ADMA plasma concentration of patients before recommending arginine supplementation as a therapeutic strategy. Higher plasma concentrations of ADMA indicating that arginine supplementation would be more successful at increasing endothelial NO production and leading to beneficial effects.

Revised manuscript Lines: 1084-1087 now states:

In the future we hope to explore [^3^H]-arginine and [^3^H]-ADMA transport across the blood-brain and blood-CSF barriers in more detail determining the pharmacokinetic constants and the identity of the transporters involved. This may further contribute to understanding the contribution of ADMA efflux to any potential benefits of arginine supplementation.

7. **REVIEWER 2 comment: Statistical Analysis: The authors should provide more details about the statistical analysis used in their study. This would enhance the rigor and reproducibility of their findings.**
**AUTHOR RESPONSE:**

We apologise for this oversight.

a) We have now briefly summarised the type of tests used in the statistics section within the methods. **Line 366-369 now states:**

The following statistical tests were applied as specified in the following sections: unpaired Student’s t-test, paired Student’s t-test, One-way ANOVA with Dunnett’s multiple comparison of means or two-way ANOVA with Tukey’s multiple comparison test as appropriate.

b) Where appropriate, we have now added the type of statistical test to all the figure legends in the main text and the supplementary figures.

c) We have also added more information about the statistical tests used in the results text (see tracked changes).

d) We have also updated Figure 9, S6 Figure and S7 Figure to reflect changes in the degree of significance.

e) We have also added an additional statistical comparison to Figure S11 (see Bars).

**8. Data Availability: The authors should clearly state the availability of their data, including the raw data and any supporting information. This would promote transparency and allow other researchers to replicate and build upon their findings.**

**AUTHOR RESPONSE:**

**Lines 1089-91 (revised version): Now states:**

**DATA SHARING:**

All data underlying the results are available as part of the article and in the supporting information file and no additional source data are required.

**9. REVIEWER 2 comment: Ethical Considerations: The authors should address any ethical considerations related to their study, particularly if they involved animal models. This would demonstrate their commitment to responsible research practices.**

**AUTHOR RESPONSE:**

We have now amended the animals and anaesthesia section as follows:

**Revised manuscript Lines 143-173:**

All experiments requiring the use of animals were performed in accordance with the Animal (Scientific Procedures) Act, 1986 and Amendment Regulations 2012 and with consideration to the Animal Research: Reporting of *In Vivo* Experiments (ARRIVE) guidelines. The study was approved by the King’s College London Animal Welfare and Ethical Review Body and performed under license: 70/6634.

All animals used in procedures were adult male BALB/c mice (between 23 g and 25 g) sourced from Harlan Laboratories, Oxon, UK, unless otherwise stated. Animals were handled with due care and consideration to minimise stress. All animals were maintained under standard temperature/lighting conditions and given water and food *ad libitum*. Welfare was monitored daily by animal technicians. Animals which were showing signs of distress (such as not feeding or drinking) were brought to the attention of the named veterinary surgeon and the animal euthanized. Mice were used for all the procedures under a non-recovery anaesthetic and represent the animals of lowest neurological sensitivity to which

h the protocols can be successfully applied. Animals were weighed immediately before the administration of the anaesthetic to ensure correct dosage of anaesthetic. Domitor® (medetomidine hydrochloride) and Vetalar® (ketamine) were both purchased from Harlan Laboratories (Cambridge, UK). All animals were terminally anaesthetised (2 mg/kg Domitor® and 150 mg/kg Vetalar® injected intraperitoneally), and a lack of self-righting and paw-withdrawal reflexes (as surrogate indicators of consciousness) thoroughly checked prior to and during all procedures. If needed, additional anaesthetic was administered via the same route. During the general anaesthesia no adverse effects were expected or observed. At the end of the *in situ* brain perfusion procedure the animal was killed by decapitated under non-recovery anaesthesia. Animals were heparinised with 100 units heparin (in 0.9% m/v NaCl_(aq)_, Harlan Laboratories; Oxon, UK), administered *via* the intraperitoneal route prior to surgery. Experiments were performed between 9 am and 5 pm.

**10. Reviewer 2: Clarity and Conciseness: The authors could improve the clarity and conciseness of their writing. This would make their findings more accessible to a wider audience.**

**AUTHOR RESPONSE:**

We have used the Microsoft word editor function to identify and improve spelling, grammar, clarity and conciseness issues.

We have made minor changes throughout the document to improve clarity and conciseness. These changes are tracked.

**11. Reviewer 2: Please improve the picture images of all, as the image is not in the higher pixel, and the image is getting blurred.**

**AUTHOR RESPONSE:**

We have improved the quality of all the figures in the manuscript and the supporting information file

**Request by PLOS ONE**

Further to your e-mail of 13.9.2024 we have now added additional information in the manuscript to provide more details on:

(1) **Methods of sacrifice:**

**We already state on line 156:** Mice were used for all the procedures under a non-recovery anaesthetic and represent the animals of lowest neurological sensitivity to which the protocols can be successfully applied.

**We now mention**

**Line 161:** All animals were terminally anaesthetised.

**Line 165:** At the end of the *in situ* brain perfusion procedure the animal was killed by decapitated under non-recovery anaesthesia.

**(2) methods of anaesthesia or analgesia, and**

**We now mention**

**Line 158:** Animals were weighed immediately before the administration of the anaesthetic to ensure correct dosage.

**Line 164:**

If needed, additional anaesthetic was administered via the same route.

(3) **efforts to alleviate suffering.**

**We now mention**

**Line 151:** Animals were handled with due care and consideration to minimise stress.

**Line 165:** During the general anaesthesia no adverse effects were expected or observed.
